# Analysis of aberrant methylation on promoter sequences of tumor suppressor genes and total DNA in sputum samples: a promising tool for early detection of COPD and lung cancer in smokers

**DOI:** 10.1186/1746-1596-7-87

**Published:** 2012-07-20

**Authors:** Leda Guzmán, María Soledad Depix, Ana María Salinas, Rosa Roldán, Francisco Aguayo, Alejandra Silva, Raul Vinet

**Affiliations:** 1 Departamento de Bioquímica, Facultad de Ciencias, Pontificia Universidad Católica de Valparaíso, Valparaíso, Chile; 2 Facultad de Salud, Escuela de Tecnología Médica, Universidad Santo Tomás, Santiago, Chile; 3 Unidad de Enfermedades respiratorias, Hospital San José, Santiago, Chile; 4 Programa de Virología, Instituto de Ciencias Biomédicas (ICBM), Facultad de Medicina, Universidad de Chile, Santiago, Chile; 5 Facultad de Farmacia, Universidad de Valparaíso, Valparaíso, Chile; 6 Centro Regional de Estudios en Alimentos Saludables (CREAS), Valparaíso, Chile

**Keywords:** DNA methylation, Sputum, Lung cancer, COPD

## Abstract

**Background:**

Chronic obstructive pulmonary disease (COPD) is a disorder associated to cigarette smoke and lung cancer (LC). Since epigenetic changes in oncogenes and tumor suppressor genes (TSGs) are clearly important in the development of LC. In this study, we hypothesize that tobacco smokers are susceptible for methylation in the promoter region of TSGs in airway epithelial cells when compared with non-smoker subjects. The purpose of this study was to investigate the usefulness of detection of genes promoter methylation in sputum specimens, as a complementary tool to identify LC biomarkers among smokers with early COPD.

**Methods:**

We determined the amount of DNA in induced sputum from patients with COPD (n = 23), LC (n = 26), as well as in healthy subjects (CTR) (n = 33), using a commercial kit for DNA purification, followed by absorbance measurement at 260 nm. The frequency of CDKN2A, CDH1 and MGMT promoter methylation in the same groups was determined by methylation-specific polymerase chain reaction (MSP). The Fisher’s exact test was employed to compare frequency of results between different groups.

**Results:**

DNA concentration was 7.4 and 5.8 times higher in LC and COPD compared to the (CTR) (p < 0.0001), respectively. Methylation status of CDKN2A and MGMT was significantly higher in COPD and LC patients compared with CTR group (p < 0.0001). Frequency of CDH1 methylation only showed a statistically significant difference between LC patients and CTR group (p < 0.05).

**Conclusions:**

We provide evidence that aberrant methylation of TSGs in samples of induced sputum is a useful tool for early diagnostic of lung diseases (LC and COPD) in smoker subjects.

**Virtual slides:**

The abstract MUST finish with the following text: **Virtual Slides** The virtual slide(s) for this article can be found here: http://www.diagnosticpathology.diagnomx.eu/vs/1127865005664160

## Background

Chronic obstructive pulmonary disease (COPD) is a cluster of heterogenic disorders, characterized by expiratory flow limitation [1] chronic bronchitis and emphysema [2-5]. The impact of COPD in the world population is significant; according to a study of the World Bank and the World Health Organization, COPD affects 210 million people worldwide, and if the current trend remains unchanged, by 2020 COPD will be the third cause leading to death in the world [6]. Cigarette smoke is the most commonly encountered risk factor for this disease [6], as well as in the development of a COPD co-morbidity, the lung cancer (LC) – the deadliest cancer in men and women around the world [7], [8]. Several studies have shown that smokers who develop COPD have a higher risk to develop LC than no smokers [9], [10].

Since early detection of COPD is an almost impossible task in asymptomatic subjects, and the radiologic and cytological methods employed for detection of LC lack sensitivity and accuracy in early stages of the disease [11], [12], the diagnosis is late in most cases, resulting in a low overall survival rate for LC (<15%) [11].

Therefore, the development of methods based in the analysis of new diagnostic markers, with high sensitivity and prognostic value for early detection of COPD and LC, in high-risk subjects (chronic smokers with GOLD 0), will be beneficial to improve quality of life – there is evidence that smokers diagnosed with COPD are more successful in quitting [13] – and to prevent the further development of LC, reducing in this way the mortality of both lung diseases [13], [14].

**Figure 1:**
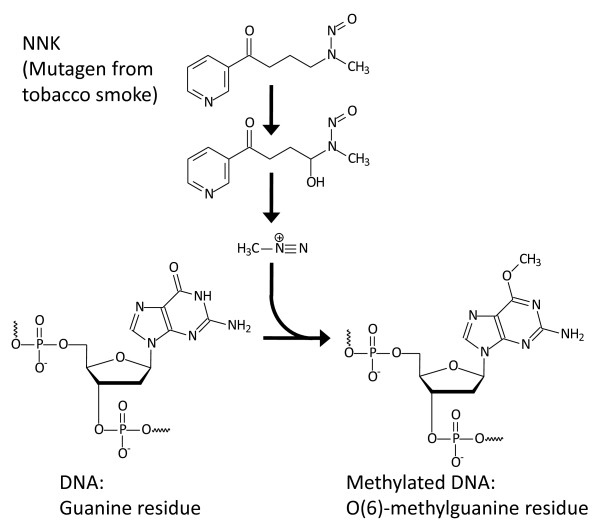
The tobacco specific nitrosamine 4-(methylnitrosamine)-1-(3-pyridyl)-1-butanone (NNK), and the proposed mechanism of guanosine methylation in DNA.

There is strong evidence towards about key role of DNA methylation and mutation events in airway epithelial cells in the early development of COPD. Some molecular alterations in DNA are produced by reactive oxidant species (ROS) found in tobacco smoke [15], [16]: e.g. the tobacco specific nitrosamine 4-(methylnitrosamine)-1-(3-pyridyl)-1-butanone (NNK) is a precursor of the alkylating agents leading to the methylation of guanosine residues in DNA [17] (Figure 1).

The inflammation in the lung airway resulting from those molecular alterations may contribute to COPD pathogenesis and may predispose to the development of LC [18], [19]. Previous work in our group, carried out in Chilean subjects by methylation-specific PCR (MSP), demonstrated a high prevalence of CDKN2A promoter methylation in squamous cell lung carcinomas (SQCs) and adenocarcinomas (AdCs) [20], [21]. Although the whole pattern of DNA methylation in bronchial epithelial cells associated with COPD is poorly known, the methylation of the afore mentioned promoters has been described in sputum, bronchial epithelium and brush biopsy of active and former smokers, subjects at high-risk for developing LC [19], [22].

On the other hand, previous studies have shown that: i) cancer patients have a higher amount of cell-free DNA in plasma, serum, sputum and other body fluids compared to healthy subjects [23], [24], and; ii) many active and former smokers have increased bronchial secretions (sputum) containing exfoliated cells (from the lower respiratory tract) [24].

Inspired in the above results we propose non-invasive methodology for early detection of COPD on smokers at risk (stage GOLD 0) to develop this disease, as well as LC, based on: i) the quantification of the amount of DNA in sputum samples, and, ii) the detection the methylation pattern of gene-promoters of TSGs critical to tumor suppression – cyclin-dependent kinase inhibitor 2A (CDKN2A, encoding p16^INK4a^ protein regulator of progression through the G1 phase of the cell cycle [25]), MGMT, encoding the O(6)-methylguanine-DNA methyltransferase (a key DNA-repairing enzyme for removal of methyl group from mutagenic O(6)-methylguanine [26]), and CDH1 (encoding the H-cadherin, key for intracellular adhesion [27]).

In this paper we show the results of the analysis of sputum samples from patients with COPD, LC, as well as in healthy subjects (total DNA and frequency of CDKN2A, CDH1 and MGMT promoter methylation), and how them correlate with the development of COPD and LC. Regarding the significant impact of COPD and smoking in Chile, – 18,000,000 inhabitants, 1.6 millions of COPD patients [28] and 4,800,000 smokers [29] – this paper is the first step in the development of an useful tool to detect, control and prevent the lung destruction and the development of LC in our country.

## Materials & methods

### Subjects and specimen collection

All the subjects included in this study were diagnosed as COPD, LC or healthy (CTR) between 2006–2007, at Hospital San José, Santiago, Chile. Pulmonary function was assessed by a screening spirometry, defined by the Global Initiative for Obstructive Lung Disease (GOLD), to diagnose and classify COPD level in all subject included in this study [13]. The subjects enrolled were divided into three groups: Group of 26 subjects with LC - diagnosed by chest-X and histopathological examination of primary tumor biopsies obtained during surgical resection [30]. According to the above tests, the malignancies in this group were classified as NSCLCs (N = 25), adenocarcinoma AdCs (N = 7), SQCs (N = 5), large cell lung cancer (LCLC) (N = 13) and small cell lung carcinoma (SCLC) (N = 1). Group of 23 patients with COPD (stages GOLD 0–4): 16 smokers and 7 former-smokers. Group of 33 non-smokers healthy subjects (CTR).

All enrolled subjects filled out a questionnaire, providing information relevant to the study: familiar history, occupational exposure, and other diseases. The clinical pathological data of each subject was followed for a period of 5 years. All demographics and clinic pathological features of the patients used in this study are show in the Table 1. The study was approved by Ethics Committee of Santo Tomás University Board and San José Hospital and informed consent was obtained from all subjects.

**Table 1:** Demographic and Clinic-pathological features of the subjects under study

***Variable***	***CTR***	***COPD***	***LC***
	***(N = 33)***	***(N = 23)***	***(N = 26)***
Age (y)	55 (23–82) ^2^	66 (41–85)^2^	66 (25–87)^2^
Male (%)	15 (45)	10 (44)	18 (69)
Female (%)	18 (54)	13 (56)	8 (31)
Smoking history (%)			
Never smoker	33 (100)	0 (0)	8 (31)
Ex-smokers	0 (0)	7 (30)	7 (27)
Smokers	0 (0)	16 (70)	11 (42)
**Smoking Frequency (%)**			
< 35 cigarette pack	0	18 (78)	10 (56)
≥ 35 cigarette pack	0	5 (22)	8 (44)
**COPD by Spirometry (%)**			
GOLD 0	-	4 (17)	7 (27)
GOLD 1	-	5 (22)	1 (4)
GOLD 2	-	5 (22)	12 (46)
GOLD 3	-	8 (35)	5 (19)
GOLD 4		1 (4)	1 (4)
Histology (%)	-	-	
NSCLCs	-	-	25 (96)
-SCC	-	-	6 (23)
-AD	-	-	6 (23)
-LCLC	-	-	13 (50)
SCLC			1 (100)
**Cytological analysis (%)**			
Normal cells	29 (88)^3^	5 (22)	8 (31)
Inflammatory cells	4 (12)	18 (78)	3 (12)
Atypia cellular^4^	-	-	6 (23)
CIS (cancer cells)^5^	-	-	12 (46)
Sputum DNA (ng/mL)	2.75 (0.00-4.95)^6^	15.95 (8.25-20.35)^7^	20.35 (4.95-28.60)

^1^ Statistical comparisons are between cases (COPD, LC) and CTR.

^2^ Median (minimum and maximum).

^3^ p < 0.05 compared with COPD.

^4^ Atypia cells (was classified from mild to severe.

^5^ CIS = *Carcinoma in situ.*

^6^ p < 0.0001 compared with COPD and LC.

^7^p > 0.05 compared with LC.

All patients and the control subjects underwent sputum induction, using the ultrasonic nebulization technique previously described [31]. Briefly, inhalation time was 2 min, followed by oral cleaning with water to avoid contamination with postnasal drip or saliva and drying of the mouth, and subsequent sputum expectoration over a period of 3 min. The procedure was repeated for at least 5 times. Sputum samples were collected separately, immediately placed on ice and stored at −80°C until use. In addition, two slides from sputum samples without proteinase K treatment were prepared and underwent Pap procedure to cytological analysis by cytopathologist [32].

### Cytology examination

The cytological analysis from slides involving a microscopic examination of exfoliated cells that are coughed up from the lungs, to determine presence of atypical cells: normal cells, tumor cell and inflammatory cells.

### DNA extraction and quantification

Total DNA was purified from sputum by digestion with proteinase K (200 g/ml) at 50°C for 1 h and vigorously mixed by vortex, followed by extraction using Wizard DNA genomic kit (Promega Corp., Madison, WI), following the instructions of the manufacturer. The DNA was quantified by absorbance at 260 nm and the purity was estimated from the ratio of absorbance at 260 and 280 nm. All the quantifications were performed in triplicate. The suitability of each sample for PCR was determined by the successful amplification of a fragment of the beta-globin gene, as previously described [33].

### Analysis of methylation by specific PCR (MSP)

To determine the methylation status within the promoter region of the genes under study, 1 μg of purified genomic DNA from each specimen was used. The DNA was denatured with 0.3 M sodium hydroxide and modified with 2.5 M sodium bisulfite (Sigma Chemical Co., St Louis, MO), as described previously [34]. The modified DNA was purified using Wizard Cleanup system (Promega Cor., Madison, WI). To amplify CDKN2A (GenBank: NG_007485.1), MGMT (GenBanK: NC_000010) methylated and non-methylated regions, a nested MSP protocol was used as previously described [34], [35]. Briefly, to amplify CDKN2A and MGMT promoter fragment in the first PCR (outer primers), 2 μL of modified DNA were used and the reaction mixture contained 1X PCR buffer, 0.1 mM dNTPs, 2 mM MgCl_2_, 0.2 μM each primer, 1.25 U Taq Gold DNA polymerase (Applied Biosystems, Foster City, CA) in a final volume of 25 μL. The PCR conditions were initial denaturation at 94°C for 10 min followed by 40 amplification cycles consisting of: denaturation at 94°C for 30 s, annealing at 66°C for 30 s, (CDKN2A) or 52°C (for MGMT) and a final extension at 72°C for 30 s. A second PCR round using inner methylated and unmethylated specific primers and 40 cycles of amplification was made using 1 μL of the first PCR product. The annealing temperature was adjusted to 72°C (for CDKN2A) and 62°C (for MGMT). In order to amplify the CDH1 (GenBanK: NG_008021) promoter fragments, PCR amplification using primers for methylated and unmethylated genes was done using 2 μL of modified DNA as template, as previously described. The reaction mixture contained 1X PCR buffer, 0.25 mM dNTPs, 2 mM MgCl_2_, 1.0 μM each primer, 1.5 U Taq Gold DNA polymerase (Applied Biosystems, Foster City, CA) in a final volume of 25 μL. The PCR conditions were: initial denaturation at 94°C for 5 min, 40 amplification cycles of denaturation at 94°C for 30 s, annealing at 57°C for 30 s, (for CDH1) and a final extension at 72°C for 30 s. The methylation status of CDKN2A, MGMT and E-cadherin promoter region was determined by analysis of PCR products into non-denaturing 10% polyacrylamide gels and 3% agarose gels stained with silver or ethidium bromide, respectively. Genomic DNA from peripheral blood treated with SssI methyltransferase (New England Biolabs, Beverly, MA) was used as positive control. Negative controls without DNA were included.

### Statistical analysis

We used Fisher’s exact test to compare frequency of variables between different groups. Odds ratios (OR) were calculated with 95% confidence intervals (CIs). The distribution of continuous variables that had skewed distribution was summarized with medians and ranges and differences between groups were tested using the nonparametric Kruskal-Wallis test and Dunn's multiple comparison post test. A P value less than 0.05 was considered statistically significant. Statistical analyses were performed using Prism 5.0 (GraphPad Software Inc., USA). Logistic regression was run using SPSS statistical software package for Microsoft Windows (version 17.0, SPSS Inc., USA).

## Results

Table 1, shows a summary of demographic and clinicopathological features of patients and CTR. Nineteen of the 26 LC patients had COPD and 7 presented normal spirometry. The GOLD stages are indicated in the Table1. Twenty-three smoker and ex-smoker were diagnosed with COPD; 4 were classified as GOLD 0 and 19 were classified as GOLD (stages 1 to 4). Thirty-three CTR, presented normal spirometry. All patients were diagnosis between 2006–2007 years.

The cytological examination was carried out in samples from all patients (COPD and LC) and CTR, as indicated in Table 1. Malignant cells were present in sputum samples of 12 LC patients (46%), while 6 cases presented mild to severe atypia. Normal cells were present in sputum samples of 8 LC patients. The COPD patients (smokers and ex-smokers) had an increased inflammatory cell numbers (neutrophil and macrophages) compared with CTR group without COPD (78% *vs* 12%).

### Analysis of sputum DNA concentration

**Figure 2:**
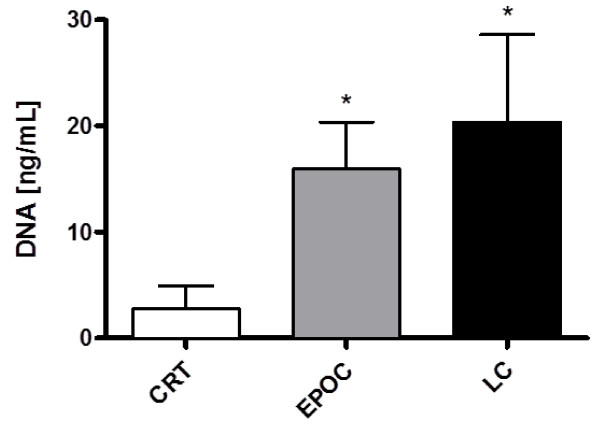
**Representative analysis DNA of concentration from sputum samples from CTR, COPD and LC subjects.** DNA concentration in samples of induced sputum obtained from COPD, LC and CTR subjects. Columns show the median and range values for each data group. * P < 0.0001 respect to control group (CTR), ** p >0,05 respect to COPD group.

The amount of DNA extracted from sputum ranged from 0.00 to 4.95 ng/mL, 8.25 to 20.35 ng/mL and 4.95 to 28.60 ng/mL for CRT, COPD and LC groups, respectively (as indicated in Table 1 and Figure 2). Differences in DNA concentration in sputum samples were significant when CTR group was compared to COPD and LC group (p < 0.0001). Thus, LC group had 7.4-fold increase in DNA concentration compared to CRT group. Similarly, COPD group had 5.8-fold increase in DNA concentration compared with CTR group (p < 0.0001). The concentration of DNA from sputum of patients with COPD and LC did not show significant differences (Figure 2).

### Analysis of gene promoter methylation in induced sputum

**Figure 3:**
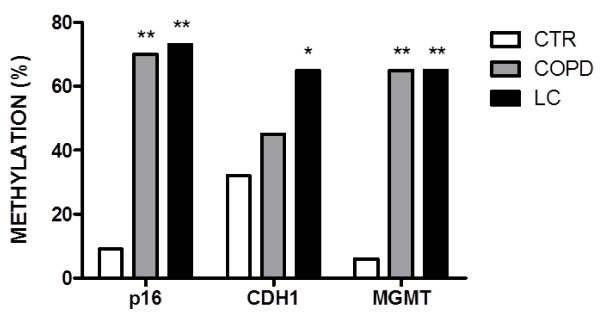
**Representative analysis of methylation promoter frequency from sputum samples from CTR, COPD and LC subjects.** Methylation promoter frequency (%) for p16, CDH1 and MGMT in COPD and LC group, both compared with CTR group. Group differences were assessed using Fisher’s test. ** P < 0.0001 respect to control group (CTR). * P < 0.05 respect to control group (CTR).

The DNA extracted of all samples under study was suitable for MSP analysis, according to the test of amplification of positive the 110 bp betaglobin gene fragment. The frequency of gene promoter methylation of CDKN2A, MGMT and CDH1 genes in sputum of patients and CTR is shown in Figure 3. In COPD patients, the prevalence of CDKN2A and MGMT methylation showed a statistically significant difference compared with CTR (p < 0.0001). Only the CDH1 gene showed a non-statistically significant difference as indicated in Table 2. The frequency of methylation was notably similar between COPD and LC patients, for the three genes evaluated, showing a non-statistically significant difference (Figure 3, Table 2).

**Table 2:** Prevalence and odds ratios for gene promoter hypermethylation in sputum

***Gene***	***CTR***^***1***^	***COPD***					
	***No. Pos (%)***	***No. Pos (%)***	***OR (95% CI)***	***P***	***LC No. Pos (%)***	***OR (95% CI)***	***P***
P16	3 (9)	16 (70)	22.9 (5.2-100.7)	0.0001	19 (73)	27.1 (6.2-118.0)	0.0001
CDH1	10 (32)	10 (45)	1.8 (0.6-5.4)	0.3950	9 (35)	4.0 (1.3-12.0)	0.0173
MGMT	2 (6)	15 (65)	29.1 (5.5-154.1)	0.0001	17 (65)	29.3 (5.7-151.4)	0.0001

Pos: Positive.

^1^Reference Group.

**Figure 4:**
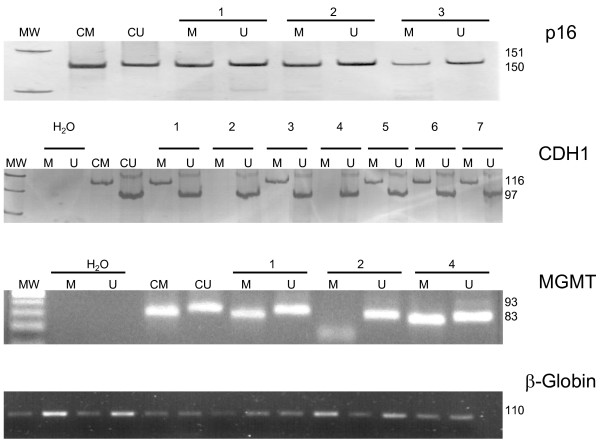
**Representative examples of MS-PCR analysis of 82 induced sputum clinical samples.** Bisulfite-modified DNA was amplified with specific primers to detect methylated DNA (M) and unmethylated DNA (U); MW: Molecular weight marker; *in vitro* genomic DNA treated with SssI enzyme used as positive control for methylated genes. (CM); peripheral blood lymphocytes DNA used as a control of unmethylated genes (CU); H_2_0: distilled water used as negative control in the PCR amplification.

The OR in the sputum samples ranged from 1.8 to 29 for detecting promoter methylation in a specific gene evaluated in COPD group compared to CTR group (Table 2). In the case of LC group the OR for each specific gene were of 4.27 and 29.3, respectively (Table 2). A representative MSP for clinical samples of COPD, LC and controls is shown in the Figure 4.

### Relationship between methylation, smoke exposure and LC risk

**Figure 5:**
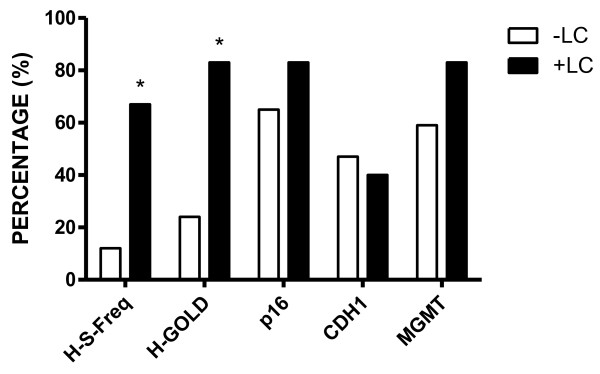
**Biomarkers obtained in COPD patients evaluated in 2012 as negative LC (−LC) and positive LC (+LC).** Figure shows as a percentage H-S-Freq (High Smoke Frequency), H-GOLD (High GOLD: GOLD > 2) and methylation on p16, CDH1 and MGMT. * P < 0.05 respect to -LC group.

Figure 5 shows as a percentage H-S-Freq (High Smoke Frequency), H-GOLD (High GOLD: GOLD > 2) and methylation on p16, CDH1 and MGMT. * P < 0.05 respect to -LC group Biomarkers obtained in COPD patients evaluated in 2012 as negative LC (−LC) and positive LC (+LC). After five years of COPD clinical diagnosis, 6/26 (23%) patients developed cancer: one had normal spirometry (GOLD 0) at the time of diagnosis, four had GOLD 3 and one GOLD 4. Four patients were smoker with smoking frequency of 35 packs of cigarettes/year and only one patient quit smoking in the five years before 2012. As depicted in Figure 5, only H-S-Freq and H-GOLD showed statistically significant difference between + LC and –LC patients.

## Discussion

The goal of study was verify our hypothesis and assess the usefulness of TSGs promoter methylation and total DNA measurement in sputum samples, as biomarkers of early stages of COPD as GOLD 0. Our results showed an increase of amount of DNA in sputum samples from smokers or ex-smoker with lung diseases (COPD or LC), compared with control subjects never-smoker (CTR). However, if the correlation between total DNA in the sputum sample and occurrence of LC is not clear. Although there is evidence that patients with cancer have larger amounts of DNA in the septum compared with non-cancer patients [36]. The DNA may have several sources: airway epithelial cells, macrophages, inflammatory cells and in some cases, only a minor fraction from tumor cells [37], [38]. The patients with cancer have larger amount of DNA circulating in plasma or serum compared to healthy subjects; probably the DNA is released into circulation after cell lyses triggered by necrosis or apoptosis during tumor development [37], [38]. Our results showed a higher DNA concentration in sputum from LC and COPD patients compared with CTR group (15.95 to 20.35 vs 2.75 ng/mL). All patients with COPD and without cancer, at the moment of diagnosis were classified as GOLD 0 to 4 by spirometry. Six COPD patients developed cancer after five years from the initial COPD diagnosis at 2006–2007.We also evaluated the frequency of CDKN2A, CDH1 and MGMT promoter methylation in sputum samples of the groups under study. Few studies show the feasibility to use DNA from sputum samples, for the detection of molecular alterations [23]. In this study, we confirmed the aberrant methylation of CDKN2A and MGMT promoter regions in LC (73% and 65%, respectively) and COPD (70% and 65%, respectively) compared with CTR (9% and 6%, respectively). There was no significant difference of aberrant methylation at CDH1promoter between LC (35%), COPD (40%) patients and CTR (32%). We found a high percentage of CDKN2A and MGMT promoter methylation in COPD and LC patients, observing a non-statistically significant difference in both pathologies. The high frequency of promoter methylation had strong association with high smoke frequency present in all patients studied. Previously, several studies have shown a variable frequency of gene promoter methylation in LC: CDKN2A (range: 23 -70%), CDH1 (range: 40-50%), and MGMT (range: 16-47%) [39-41]. Discrepancies in the rates of methylation are mainly due to the use of different specimens (fresh tumors, paraffin-embedded tissue, cell lines, bronchioalveolar lavage and sputum) containing a diverse DNA concentration or different analytical approaches [41-43]. Recently, Belinsky *et al.* (2006) reported the feasibility to use the sputum to determine the promoter methylation in 14 genes [42]. However, the cohort used in this study all LC patients and control had subjects a smoking history of ≤50 packs/year (current and former). In this report, all control subjects showed aberrant promoter methylation in all the genes studied. In addition, was demonstrated that CDKN2A and/or MGMT promoter methylation can be detected in DNA obtained from sputum in 100% of patients with SQC up to 3 years before clinical diagnosis [43]. A similar frequency of CDKN2A promoter methylation was observed in our study, as indicated above. Our results, confirmed previous findings using a different study group: the methylation of CDKN2A and MGMT were more frequent in smoker patients with LC compared with never smokers [44], [45]. Nevertheless, our results about the frequencies of aberrant promoter methylation of CDKN2A and MGMT genes between smokers and never-smokers do not agree with another previously reported [45]. The explanation for the observed discrepancies among different studies is unclear. However, we can speculate that these discrepancies are due to the small number of never-smokers involved in these studies [45-47]. In our report, only eight patients with LC were never-smokers during their lifetime. A key element of our analysis is the meaning of “never-smoker”. A never-smoker is a patient who smoked no cigarettes during his lifetime, also discussed previously [46]. However, in several studies the never-smoker has been defined as a patient who has smoked less than 100 cigarettes [47-49]. The effect of the different analytical approaches for DNA purification from sputum, employed in previous studies cannot be ruled out. Also determined, that he frequency of promoter methylation of CDKN2A and MGMT was associated with a 22.9 and 29.1-fold increase in the risk to develop cancer in smoker COPD patients. This result is interesting, since Belinsky *et al.,*[42] reported only 6.5-fold increase in the risk of LC development, through detection of three o mores genes methylated in the sputum. In our cases, both smoking frequency and aberrant promoter methylation of CDKN2A and MGMT gene were associated with progression of COPD diseases in the group of patient without LC. Patients with GOLD > 2 diagnosed as negative to LC in the 2007 developed LC at the beginning of 2012. Other patient with GOLD 0 at the time of diagnosis has LC actually have been GOLD 1 by spirometry. All this patients have smoked between 20 and 35 packs of cigarettes/year for over 40 years during his life. Therefore, these results show a positive correlation between H-S-Freq and H-GOLD with a high prevalence to develop LC.

Although in this study not investigated the effect of cigarette smoking on cellular structures of the bronchial airway. We found have increased sputum inflammatory patterns (neutrophil and macrophage) numbers compared with subjects without COPD nonsmoker. This study showed that there are no significant differences in the cellular profile in sputum samples between patients with COPD who are active smokers and those who have ceased smoking. Similar observation has been demonstrated in other studies [50].

It is known that gene promoter methylation is a very early event in tumoral progression, so our findings probably suggest and confirm that molecular alterations as methylation occur in a temporal-dependent manner during a long time before COPD arising or LC development. A recent review [19], described that oxidants generated endogenously, such as air pollutants or cigarette smoke, generate ROS, producing an inflammatory response in lung cells by activation of stress kinases as c-Jun or NF-Kβ and phosphoinositide-3 kinase (PI-3 K) and other signal transduction pathways, leading to enhanced gene expression of pro-inflammatory mediators [51]. Recently, Damiani *et al.*[52] have proposed that epithelial molecular alterations in bronchial cells - such as an increase of DNA methyltransferase expression, histone modification, DNA methylation and p53 and K-ras genes mutations - are induced by tobacco carcinogens. These epigenetic and genetic alterations deregulate the expression of genes involved in the anti-inflammatory response and block the DNA repair, contributing to malignant transformation [53], [54]. The involvement of environmental factors – e. g: pollution exposition - in the TSGs methylation pattern observed in our study cannot be ruled out. In fact, it has been reported that pollutant exposure in modern-cities, is a major factor leading to aberrant promoter methylation of TSGs [55]. Therefore, tobacco smoking is a necessary but not sufficient condition for LC development; the synergistic effect between smoking and environmental pollutants must be studied in the future.

Finally, the most significant factor for survival in lung cancer is the stage of disease at diagnosis. Therefore, our results suggest that DNA present in the sputum is an useful non-invasive sample to identify epigenetic alterations such as gene-promoter methylation. These results highlight a potential value of this MSP-sputum molecular marker for early detection of LC in high-risk individuals as smoker at risk (GOLD 0), and may be a complement to current diagnostic techniques used to diagnosis of lung diseases.

## Competing interests

The authors declare that they have no competing interests.

## Authors’ contributions

LG carried out the experimental studies and drafted the manuscript. MD, AMS and SA carried out the molecular analysis. RV provided the body fluid samples and clinical data of the patients. LG and FA designed and coordinated the study. RV performed the statistic analysis of the dates. LG and RV contributed in the preparation of the draft version and manuscript review. All authors reviewed the draft manuscript and approved the final version for submission.
